# Incorporation of Additives and Fibers in Porous Asphalt Mixtures: A Review

**DOI:** 10.3390/ma12193156

**Published:** 2019-09-27

**Authors:** Anik Gupta, Jorge Rodriguez-Hernandez, Daniel Castro-Fresno

**Affiliations:** GITECO Research Group, University of Cantabria, 39005 Santander, Spain; jorge.rodriguez@unican.es (J.R.-H.); castrod@unican.es (D.C.-F.)

**Keywords:** permeable pavement systems, porous asphalt mixtures, fibers, additives

## Abstract

Despite the numerous benefits for preserving the hydrological cycle, permeable pavement systems (PPSs) found their major application in parking spots and for light traffic scenarios due to their limited durability and strength. To make the PPSs suitable for heavy traffic conditions without significant distresses, research is shifting toward the adoption of novel binders and additives for designing multifunctional porous asphalt mixtures which make up the surface course of PPSs. Certain additives are well known for enhancing the durability of dense graded asphalt mixtures and improving fatigue and rutting resistance. However, the studies on the influence of additives on abrasion resistance and binder draindown, which are the common problems in porous asphalt mixtures (PAMs), are still not well established. This paper summarizes best practices performed on PAMs and recommends possible future research directions for its improvement. Particular emphasis is placed on strength and resilience of PAMs by incorporating additives like nanosilica, crumb rubber, warm-mix additives, fibers (such as cellulose, glass, steel, and synthetic fibers), and some eco-friendly materials. It was found that different additives seem to have different effects on the properties of PAMs. Moreover, the combination of additives has synergistic benefits for the performance of PAMs, especially in urban pavements.

## 1. Introduction

Due to soil sealing, large amounts of runoff lead to ponding in urban areas and erosion in the unsealed neighboring areas [1]. The present drainage systems have limited capacity to hold runoffs, thus leading to urban flooding problems during overflow. Floods are now more frequent and have greater impacts on the livelihood of society. Even in regions of high rainfall, lowering of groundwater due to soil sealing resulted in scarcity of water for plants and vegetation. an increase in impervious surface areas also reduced the ability of soil to filter water, which increased the amount of impurities and contaminants entering the water cycle [2]. In this situation, it is vital to spread the idea of building sustainable systems with the ability to restore balance in the natural ecosystem.

Sustainable urban drainage systems (SUDS) are structures that imitate the drainage pattern of natural land surface. Types of SUDS include bio-retention cells, green roofs, infiltration trenches, rain barrels, rain gardens, permeable pavements, rooftop disconnection, and vegetative swales [3]. Permeable pavement systems (PPSs) are one of the most complete SUDS and they attracted attention in previous decades due to their ability to withstand sufficient vehicular loads [4]. PPSs generate flow attenuation, which allows water to infiltrate through their structure, thereby slowing, delaying, reducing, and filtering the water flows [5,6]. Thives et al. mentioned that the water filtered through PPSs can be used for non-potable purposes like washing cars and cleaning [7]. The advantages of PPSs include reduction in tire noise, water splashing, skidding, and soil erosion. They also mitigate the urban heat island effect, and diminish the damage to pavement caused by stagnant water, while facilitating a high infiltration capacity [8,9,10]. Even the poorest performing PPSs have greater drainage capacity than normal rainstorm events [6,11,12]. The open graded structure of PPSs provides better skid resistance and, hence, more safety to road users. By replacing conventional pavements with PPSs, the number of accidents occurring in wet weather can be reduced by up to 80% [13].

The surface of the PPS is mainly constructed with porous asphalt mixtures (PAMs). In PAMs, very small amounts of fines (or none) are added to form an open-graded structure to allow water to flow through it. There is a stone-to-stone contact, resulting in higher air void content as compared to dense graded mixtures. Subsequently, due to more environment exposure, aging is faster in PAMs [14,15,16]. The constant environmental exposure increases the asphaltene content, which makes bitumen stiffer and brittle, leading to raveling and cracking [17,18]. Raveling is the most significant damage in PAMs as it spreads very quickly and results in a huge reduction in pavement life span [19]. Raveling is more prominent in winter seasons. The slowest lanes deteriorate faster than the overtaking lane and raveling is faster in old sections. Moreover, high traffic is responsible for a higher amount of raveling [20]. At the same time, due to clogging, functionality is affected, and the porous pavement is not able to facilitate drainage, reduce noise, and prevent damage due to water stagnation. The rate of clogging depends on several factors like type of materials used in the pavement such as cement concrete or asphalt, slope of pavement, maintenance measures adopted, environmental conditions, erosion rate around the surface, etc. [11,21].

Due to different vehicle loads, rainfall durations, and rainfall intensities, there are different requirements of PPSs in urban regions [4]. For instance, in residential areas, the noise reduction may be the most prime concern, whereas, for highways, skid resistance may be the most significant parameter. The development of multifunctional PPSs during urban planning requires materials that endure different loading conditions while maintaining the integrity of the structure. The idea of including additives in the mixtures, especially fibers, is relatively new in permeable pavements, as very limited literature is available. In the 2000s, the need to implement PPSs led researchers to search for ways to improve the quality of permeable pavements by incorporating various additives [22,23]. Additives like nanosilica were shown to improve the abrasion resistance of the PAM considerably [24]. Natural fibers such as cellulose fibers are among the most commonly used fibers in PAMs as they improve the binder stability [2,25,26,27,28,29,30,31]. For low-temperature crack resistance, glass fibers can be used [32]. To enhance properties such as elastic modulus and tensile strength (ITS), synthetic fibers showed promising results [33]. Acrylic fibers improve the mechanical performance under extreme conditions; thus, they can be utilized in cold and warm regions [34]. Studies also showed that recycled aggregates can also be used to replace virgin aggregates on low-intensity traffic roads [35,36]. The type of binder is an important factor, as a high-viscosity bitumen improves the cohesion of the binder. Meanwhile, polymer-modified bitumen (PMB) improves the adhesion between aggregates and the binder [16,30,37].

The objective of this paper is to address the role of different additives and fibers in improving the performance of PAMs. Subsequently, various aspects of design of porous asphalt mixture are discussed in detail. The influence of gradation adopted in different countries is discussed based on the mechanical and functional performance of the permeable pavement systems. Special emphasis is placed on the wide variety of additives that can be used for the implementation of durable permeable pavement systems for multifunctional urban applications. The mechanisms via which additives influence the properties of PAMs are elaborated in detail. Furthermore, the feasibility of using eco-friendly materials in PAMs is discussed.

## 2. Porous Asphalt Mixtures Design

### 2.1. Gradation

PAMs are made of the same materials as dense graded bituminous mixes except with fewer fine aggregates; however, their durability is significantly less. This shows that the performance of the mix depends greatly on the proportion of fine aggregates as they interlock the aggregate and binder [38]. For the mixture to have adequate strength and durability, it is necessary to have fine aggregates, but increasing the amount of fine aggregates compromises the permeability. If the air void content is less than 14%, the permeability is negligible. Therefore, the binder content increment results in a considerable reduction in vertical hydraulic conductivity and porosity, due to clogging of voids [39]. The dynamic modulus is also lower for PAMs due to higher air void content; an increment of 1.5–3 times was observed upon reducing the air void content from 20.5% to 9.5%. However, after moisture conditioning, the dynamic modulus increases in the case of PAMs, which may be explained by the pore water in the voids [40]. 

The type of filler plays an important role. Limestone is the most widely used filler in PAMs [41,42,43,44,45], whereas hydrated lime can be used as an antistripping agent as it controls moisture susceptibility; furthermore, diatomite can be used to improve adhesion and low-temperature cracking resistance [46]. Fine aggregates are allowed in the PAM design in small quantities [5,6,8,9]. In the United States (US) [47], Australia [48], and Malaysia [49], the percentages of fine aggregates are 14.5%, 14%, and 13.5%, respectively, with approximately 3% of filler content, whereas, in Singapore [50], 15.5% fine aggregates and 6% fillers are allowed in the mix. As expected, if designed according to Singapore guidelines, the permeability of PAMs will be lower but the strength will be higher in comparison to US, Australian, and Malaysian guidelines [51].

This relationship was further explored by analyzing the effect of gradation on durability, strength, and rutting of the mix. In the US [29], a study was conducted on a comparison of gradations of 10 different states. It was found that the strength varies indirectly with porosity of mix as, when the porosity of the mix increases, the indirect tensile strength (ITS) decreases. Meanwhile, it was highlighted that the permeability, which varies directly with porosity, can, therefore, be estimated at the time of gradation. Moreover, no statistical relationship was obtained between abrasion or rutting resistance with the air void content of the mix [29,39,42,52]. The rutting behavior can be explained as deformation is more dependent on the suitability of the binder and packing of aggregates than on the air voids.

Zhang et al. [53] used a homogenization technique to compute the stiffness of the stone-to-stone contact of aggregates. In this study, the Mori–Tanaka (MT) model was used. It was revealed that, at low temperatures, due to the high stiffness of the binder, the stone-to-stone skeleton is stable enough to be bonded together. On the contrary, at high temperatures, the binder is softer, which may result in a weaker network. Moreover, the stiffness of the stone-to-stone contact depends highly on the compaction effort, where a higher compaction provides a higher bearing capacity, even in high-temperature conditions. Takahashi and Partl [54] carried out a study on the effect of packing on compaction caused by traffic loads. No effect of maximum size of aggregate was observed on the air voids. However, the parameters that were more important for air voids were void size, void distribution, and packing condition. If the packing is denser, the resistance of the PAM to over-compaction due to traffic loads is improved.

### 2.2. Binder

Most commonly, polymer-modified bitumen (PMB), crumb rubber-modified bitumen (CRMB), styrene–butadiene–styrene (SBS)-modified bitumen, and high-viscosity bitumen (HVB) are used to improve the performance of PAMs. Ma et al. [25] conducted a study involving four types of binders: HVB, SBS-modified binder, PG76-22, and PG70-22. A uniaxial compressive test was performed at 15 °C under the loading rate of 2 mm/min, concluding that HVB improves the compressive strength of the PAM as compared to the SBS-modified binder. The high viscosity of bitumen improves the cohesion; thus, less draindown is observed [30]. Moreover, due to high viscosity, the stability and resistance toward abrasion also improves [25,37,38]. It is also worth mentioning here that the Cantabro test results worsen with aging of the binder, which agrees with the fact that the binder becomes stiffer and brittle due to aging, leading to more abrasion loss [30]. However, if the binder is more viscous, the asphalt mixes show more resistance to particle loss. Hence, a high viscosity of binder leads to a better mixture in terms of compressive strength, cohesion, draindown, rutting performance, and abrasion resistance.

All binders stiffen due to the aging process; however, the binder at the upper surface ages more than the lower layers even if the layers have the same air void content [55]. Polymers improve the interaction of binder and aggregates; therefore, PMB mixes are less susceptible to abrasion, moisture, and temperature changes [16,37]. However, untreated mixes have more interconnected voids; thus, the permeability of the mix is compromised while incorporating additives/modifiers [37].

Rubber-modified binders are used to increase the elasticity and lessen the thermal susceptibility of the mixtures [56,57]. Rubber improves the viscosity of the binder, which in turn enables the use of a higher binder content without the risk of binder drainage [8]. Lyons [26] used four different kinds of binder, i.e., a neat binder, a modified binder (3% SBS + PG64-22 binder), CRM 5% (neat binder + 5% CRM), and CRM 12% (neat binder + 12% CRM). The draindown was measured three times after every hour, to analyze the influence of time on the draindown. Higher percentages of CRM (12%) were required to prevent draindown in comparison to using 5% CRM. In the case of PMB, fiber was essential for preventing draindown. However, CRMB has two major limitations, one of which is the reduced permeability due to greater binder film thickness, and the other one is the requirement of high temperatures, which result in greater greenhouse gas emissions [58].

Adhesion properties of the binder are equally important in predicting the possibility of stripping in PAMs. Baldi-Sevilla et al. [59] studied the behavior of binder in unaged and aged conditions. The contact angle of the binder has a direct relationship with its mechanical resistance as, if the contact angle is high, the binder is too cohesive, and the resistance to rutting and fatigue is lower. This may be because, with less cohesion, the molecules are more likely to adjust, and the recovery is quick.

The stripping phenomenon is a thermodynamically favorable condition as, in the presence of water, the adhesion of aggregate and bitumen is worse than that of aggregate and water [60,61,62]. In the absence of fillers, using additives may improve the adhesion between aggregates and bitumen [63]. Ye and Jian [64] mentioned that the adhesion of fiber and bitumen is better than bitumen and aggregates; thus, adding fiber leads to better resistance to moisture. Although additives like diatomite have adverse effects on the adhesion of binder, they enhance the moisture resistance, which is possibly due to an increment in energy ratio (resistance to moisture increases with an increase in energy ratio). Moreover, types of aggregates also influence the adhesion, as lime aggregates show better bonding with bitumen than granite due to their larger surface area [65].

*Conclusions*: High-viscosity binder improves cohesion, which is responsible for enhanced strength, lesser draindown, better durability, and higher rutting resistance in PAM mixtures. Meanwhile, PMB and rubber-modified binders are especially effective for reducing the thermal susceptibility of mixtures. However, in the case of rubber-modified binders, high amounts are required to avoid draindown. Moreover, additives and fibers improve the adhesion properties of binder and aggregate, thereby improving the moisture susceptibility and avoiding distresses caused due to rutting and fatigue.

### 2.3. Performance Tests of PAMs

There are not many standards available for evaluating the functional performance of porous asphalt; therefore, the functionality of pavements cannot be determined during the mix design [66]. In the case of permeable pavements, functional performance is equally as important as structural performance. Tests performed to quantify the performance of the PAMs are summarized in Table 1.

## 3. Additives

### 3.1. Nanosilica

Nanotechnology received great attention around the world in past few decades. Nanosilica materials are not only abundantly available but also have a large surface area that facilitates high adsorption, high dispersion ability, and high purity [78]. Due to these factors, they are commonly used to improve the moisture susceptibility of asphalt mixes [79,80]. Nanosilica is a very stable material with low toxicity. The most appropriate dosage of nanosilica is 2–6% by weight of binder [59,72,81,82]. Silica also increases the polarity of the coarse aggregates, which subsequently improves the adhesion between aggregates and binder, which in turn reduces the particle loss due to abrasion [62]. Upon increasing the content of Nanosilica, the rut depth was found to decrease [78,81,83]. However, it is worth mentioning here that, in an open graded friction course (OGFC), rutting is not a major concern due to its thin lifts. However, in PAMs, rutting is an important parameter due to large air void presence and the tendency of aggregates to shift their location [26].

Tanzadeh et al. [81] pointed out that nanosilica in combination with fibers has synergistic benefits; on one hand, fibers improve the mechanical strength while, on the other hand, nanosilica improves various properties such as the adhesion, stability, and abrasion resistance of the PAM. Composite additives have a better effect on the properties of the PAM in comparison to adding a single additive [59,84,85]. Moreover, nanosilica increases the shear modulus and viscosity of the asphalt, which results in anti-oxidation, improved temperature stability, and better fatigue resistance in PAMs [44,81,82,83,86]. Nazari et al. [86] explained that nanosilica induces crack path deflection due to its high dispersion, arresting the propagation of cracks. A summary of the mechanical resistance of mixtures with different additives in PAMs is shown in Table 2.

### 3.2. Crumb Rubber

In CRMB, the bitumen is modified by the rubber to increase the viscosity, whereas, if used as an additive in the mix, then the rubber is added by dry process [87]. In the former case, the rubber is added in the bitumen at high temperature, when blending of rubber and bitumen is very efficient, and crumb rubber modifies the chemical connections of the binder, resulting in a more viscous modified binder. On the other hand, in the dry process, the crumb rubber (CR) is mixed with the aggregate during preparation of asphalt, and its interaction with the binder is limited. Shell handbook [87] explained its behavior as an “elastic mineral aggregate”. Eskandarsefat et al. [88], presented a new procedure to add crumb rubber with cellulose fibers to analyze the composite behavior; it was stated that rubber increases the ductility and also improves the stiffness of the mix at high temperatures. Sangiorgi et al. [57] presented a study of adding crumb rubber to PAMs. It was highlighted that, at low temperatures, the crumb rubber reduces the binder’s rigidity, resulting in higher low-temperature crack resistance. On the other hand, at high temperatures, CR improves the viscosity of the binder and, hence, binders are stiffer at high temperatures. This was further explained by Cheng et al. [71], highlighting that if crumb rubber is uniformly distributed in the binder, it improves the toughness of the binder and results in higher low-temperature crack resistance. CR also enhances the affinity of bitumen resulting in less oxidation, lower stripping, and high raveling resistance [89,90].

It was observed that the performance of the mix was dependent on the size of the CR as #20~#200 (smallest size) CR reduced the particle loss, whereas the samples with other CRs had even worse particle loss than the control mix. Moreover, increasing the rubber content led to lesser abrasion resistance. The optimum content of rubber was found to be 10% [8]. Apparently, a smaller size of the rubber can blend in the mixture more homogeneously [8,23]. Furthermore, crumb rubber reduced the ITS, moisture susceptibility, permeability, and resilient modulus of the mixture with the only exception being the #20~#200 size which improved the performance of PAM marginally. However, there was no observed effect on the permeability of the samples [23]. However, high temperatures are required for the use of crumb rubbers, which results in higher greenhouse gas emissions, and their storage may lead to sedimentation at the bottom of the container [2,87].

### 3.3. Warm-Mix Additives

Warm-mix asphalt (WMA) technology is a sustainable measure to reduce the temperature (up to 40 °C lower) for mixing and compacting asphalt pavements in comparison to the conventional hot-mix asphalt. WMA technology reduces the energy consumption and CO_2_ emissions. Moreover, using WMA technology with porous asphalt pavements can have synergetic benefits. Ranieri [91] carried out a study with a commercial warm-mix additive called Sasobit^®^ (2.5% of binder weight) in combination with cellulose fibers (0.4% by aggregate weight). Due to less compaction temperature in warm mixes, the air voids increase at the same compaction effort, which is desirable in PAMs. In cold weather, a WMA porous mix with stearic acid can be used, and, for the same dry ITS, the compaction temperature can be reduced up to 20 °C. Moreover, there is reduced aging of the binder due to a reduction in mixing temperature. However, WMA performs worse than HMA in moisture susceptibility and abrasion resistance [42,90,91,93]. In a study presented by Lastra-González et al. [42], who analyzed PAMs with CRM binder in combination with fatty-acid amide wax, Sasobit^®^ and fatty-acid amide wax were shown to have adverse effects on the cohesion of binder, reducing its resistance against abrasion and moisture susceptibility [42,91].

Frigio and Canestrari [90] found that, while using the WMA technology, the need to use stabilizing fiber was eliminated when producing similar quality PAM. The warm porous asphalt mixes outperformed HMA without fiber in terms of permeability, durability, draindown, and abrasion resistance tests. Due to a reduction in temperature in WMA technology, the aging of the binder is reduced, which improves the resistance to fatigue cracks.

*Conclusions:* Studies (Table 2) found that nanosilica enhances the binder stability, TSR, and ITS of PAMs, while crumb rubber decreases the abrasion resistance with a few exceptions [8,89] and has no effect on stiffness of the mixtures. On the other hand, warm-mix additives improve the permeability but reduce the abrasion resistance.

## 4. Additions

### 4.1. Fibers

Fibers are additions that are commonly used in conventional dense graded asphalt mixes to improve the mechanical properties [17,18,94]. The mechanism of action of fibers in asphalt mixtures is complex. Under tension, the stress is transferred from medium to fiber, and the fiber bears a portion of stress. Hence, the ability to withstand stress depends on the fiber content. Moreover, the interaction of fibers and aggregates acts as a medium for aggregates to bond together. Fibers increase the bitumen stiffness, which may lead to brittleness, responsible for distresses in the structure of the pavement [21]. In PAMs, fibers are used to prevent binder draindown and facilitate a higher optimum binder content [16,18]. The incorporation of fibers leads to a reduction in air voids in PAMs, as they interrupt the water flow through connected voids [5]. However, very limited literature is available on the use of fibers in PAMs; therefore, the influence of fibers in PAMs needs to be evaluated in detail for each particular case. Table 3 summarizes the literature review done to quantify the effect of fibers on the mechanical resistance and functionality of PAMs based on their content and type.

#### 4.1.1. Cellulose Fibers

Cellulose fibers are obtained from the bark, wood, or leaves of plants. The surface area of commonly used cellulose fibers is 10 times greater than that of mineral fibers and polyester fibers, which explains the ability of cellulose fibers to bind more binder [21]. According to the study by Ye and Jian [64], the incorporation of 0.3–0.5% cellulose fibers in an open graded friction course (OGFC) gave better stability to the binder than polypropylene and polyester fiber.

Afonso et al. [27] explained that, due to high bitumen absorption, cellulose fibers improve the rutting resistance of the PAM, whereas no improvement on raveling resistance was observed in combination with neat binders. In particular, in the case of the wet Cantabro test, cellulose fibers had adverse effects as they increased the particle loss. Regarding this, Valeri et al. [28] observed that, up to optimum fiber content, cellulose improves the raveling resistance, whereas, upon increasing further, raveling resistance reduces. As explained by the author, due to higher fiber content in comparison to binder content, the cohesiveness of the binder reduces, leading to particle loss. However, cellulose fibers in combination with modified binders improve the raveling resistance.

To account for stiffness, moisture susceptibility, and ITS, no effect was observed by adding cellulose fibers [26,27]. Lyons [26] used cellulose fibers 6 mm in length, and highlighted that cellulose fibers reduce the porosity up to 22%. If cellulose fibers are well dispersed, a high absorption of binder by cellulose fibers is observed, which may be responsible for lesser binder drainage and lesser permeability. Eskandarsefat et al. [88] found that the mixtures with cellulose fibers exhibited high Marshal stability due to the high binder absorption ability of cellulose fibers.

#### 4.1.2. Mineral and Glass Fibers

Mineral fibers are rigid fibers as they do not entangle. Mineral fibers have the lowest softening point as compared to polyester fibers and cellulose fibers. The complex modulus increases up to an optimum content. Until that fiber content, the stiffness and resistance to rutting are improved; beyond that point, the phenomenon is reversed due to fiber–fiber contact [21]. They also reduce the binder draindown of the mixes [25,81]; however, the performance of mixes with mineral fibers is worse in the case of the aged and unaged Cantabro tests. Glass fibers improve the ITS [21,82], but the moisture susceptibility resistance is reduced by adding mineral fibers [82].

Glass fibers are traditionally used in the textile processing industry. The tensile modulus of glass fibers is very high, and its thermal susceptibility is very low [82]. Therefore, a noticeable increase is observed in the ITS value of mixtures by adding glass fibers [81,82,89]. Glass fibers absorb the binder and, hence, increase the air void content and the binder content of the mix without the risk of binder drainage [94]. According to Wang et al. [32], glass fibers improve the crack resistance of asphalt mixtures, especially at low temperatures. Tanzadeh et al. [81] carried out a study with 12-mm-long glass fibers, and they reported improvements in stiffness and tensile strength. However, the fibers led to a decrease in the permeability of mix, which can compromise the water drainage ability of the PAM.

Glass fiber acts as an elastic medium to reinforce the viscoelastic characteristics of the mixture, which subsequently improves the strain value, making the mixture stiffer. Therefore, glass fibers can be added to mixtures to reduce the rut depth [89]. Moreover, glass fiber enhances the ductility, improves the fatigue strength and also preserves the high-temperature stability of the mixture by improving its rutting resistance [81,84].

#### 4.1.3. Steel Fibers

Steel fibers not only improve the mechanical performance of the fibers but also impart healing ability. They are used to heal the PAM via electro-thermal procedures. When the samples are placed in the zone of electromagnetic field, currents start flowing in the steel fibers as steel fibers are conductive in nature and warm the surrounding binder matrix. Then, the binder melts and fills the micro cracks, thus preventing crack propagation [95,96,97].

This phenomenon of self-healing is even more pronounced in PAMs. Moreover, adding steel fibers improves the resistance to particle loss considerably [97,98]. Gonzalez et al. [97] indicated that steel wool and steel grit improve the moisture susceptibility and ITS value of PAMs. At the same time, they make the mixture stiffer and more resistant to fatigue cracking. Moreover, steel fibers do not reduce the workability of the PAM, which suggests that they can be compacted at the same compaction effort. In a study by Liu et al. [96], three different types of steel fibers were used. Steel fibers of small diameter and longer length performed better than steel fibers of large diameter and shorter length, as the fiber-to-fiber contact was better in the former case, which resulted in more conductivity.

Serin et al. [98] carried out a study on different contents varying from 0% to 2.5%. The maximum stability value was observed at 0.75% fiber content. It was also found that, at higher fiber content, the stability values were worse than control samples with no fiber content, which is in agreement with the study done by Tabaković et al. [99], who explained that very long steel fibers and high contents can lead to cluster formation. Additionally, clusters can also result due to bad mixing [68]. These clusters may later become high-temperature zones which weaken the structure of the specimen. Steel fibers also improve the particle loss resistance of PAMs.

#### 4.1.4. Synthetic Fibers

Synthetic fibers are manmade fibers, prepared via the synthesis of chemicals. Polypropylene, polyester, and aramid fibers are commonly used synthetic fibers in asphalt mixes. The microstructure of polypropylene fibers is round and smooth; thus, they tend to entangle, which results in a higher softening point [21]. According to Tanzadeh et al. [81], polypropylene fibers reduced the draindown of the PAMs up to 49%. Strength was observed to be increased by up to 50% due to polypropylene fibers. Moreover, when added in combination with glass fibers, the draindown was decreased up to 80%; ITS was observed to be increased by 65% by the same combination, of which 50% of the increment was solely due to the addition of polypropylene fibers. Polypropylene fiber acts as a three-dimensional reinforcement in the mixture, which provides more binder stability and strength.

Polyester fiber improves the raveling resistance and the stability of bitumen [24,64]. Ma et al. [25] presented a study on the use of modified bitumen in combination with polyester, mineral, and cellulose fibers (2.5% by weight of aggregate). In the wet Cantabro test, mixes with polyester fibers performed well, which entails better abrasion resistance and reduced moisture susceptibility. It is worth mentioning here that the immersion time in the wet Cantabro test is an important parameter to obtain mass loss accurately. Higher immersion time leads to higher mass loss. Hence, if mixtures with polyester fibers are performing well in the wet Cantabro test with high immersion time, then fibers are improving the moisture resistance of mixtures considerably. Polyester fibers also improved the low-temperature crack resistance according to the thermal stress restrained specimen test (TSRST). However, polyester fiber has adverse effects on resilient modulus and strength, and it also compromises the permeability of the PAM [25].

The influence of addition of polyolefin and aramid fiber depends on the type of the binder, as the effectiveness of fibers is higher on the unmodified binder than on the SBS-modified binder [100]. Chen et al. [21] studied the working mechanism of mineral, cellulose, and polyester fibers in asphalt mixtures and pointed out that, due to higher interfacial area in the case of mineral fibers, mineral fibers with lower diameter have greater toughness than the polyester fibers of higher diameter. However, polyester fibers have more tensile strength than mineral fibers.

*Conclusions*: The majority of studies mentioned that all fibers improve the draindown and rutting resistance of the PAM. However, only synthetic fibers and steel fibers improve the abrasion resistance and ITS of the mixtures.

### 4.2. Eco-Friendly Materials

Eco-friendly materials are used to minimize the environmental impact of human activities as construction. They can either be materials that are recycled to save energy, or they can be materials that can reduce the consumption of natural resources. By utilizing non-toxic materials, the CO_2_ emissions can be reduced [101].

The use of waste materials such as coconut shells, coconut fibers, chitosan, date seed ash, etc. in PAM was confirmed by various studies to enhance the properties of the mix [35,36,44,45,101,102,103]. Table 4 summarizes the studies on the incorporation of eco-friendly materials in PAM.

Sangiorgi et al. [44] conducted a study on replacing limestone filler used in PAMs with bentonite, a waste bleaching clay. Results showed no significant reduction in air voids, and the ITS was increased due to addition of clay in place of limestone. Moreover, the indirect tensile stiffness modulus test showed an improvement in the stiffness of PAM using waste bleaching clay. However, the particle loss due to the addition of clay was twice as that of the reference mixture, due to different degrees of embrittlement of the mixtures. Zhang et al. [45] pointed out that a higher amount of filler-to-bitumen ratio leads to reduced particle loss and higher permeability loss independently of the type of filler added. This reduction in permeability might be explained by the blocking of air voids by adding large amounts of filler.

Chen et al. [35] presented a study on use of 100% recycled concrete aggregates (RCAs) in PAMs. It was found that RCA absorbs the binder due to its low density and higher porosity, which also results in a high abrasion value. However, the abrasion loss, draindown value, and moisture susceptibility were within permissible limits. This research was continued in [36] by using a hybrid PAM, through the addition of RCA in combination with low-utility glass. It was concluded that hybrid mixtures (78% RCA and 16% glass material) also absorb large amounts of binder and, hence, more binder content is needed for a satisfactory bonding effect. RCA performed well in dynamic creep test performance. Mohammadinia [102] utilized recycled tire aggregates in PAMs and found that the tire aggregates enhanced the stiffness of the mixture as they penetrated the interfacial bond. Therefore, RCA and recycled tire aggregates can be used in low-traffic pavements without considerably harming PAMs.

Huang et al. [103] studied the possibility of designing permeable pavement systems that absorb non-point source (NPS) pollution such as benzene, toluene, ethyl-benzene, and xylene (BTEX) by incorporation of activated carbon. Activated carbon (AC) was added at 4%, 8%, 16%, and 24% by weight in PAM to analyze the suitability as an additive. Addition of AC in the amounts 8% and 16% is recommended, as both 4% and 24% contents of AC result in higher particle loss and binder drainage. According to the estimation, the amount of removal of BTEX per km could be up to 1018, 1329, 1541, and 1670 kg, respectively, on a two-lane highway by using 8% activated carbon. Additionally, Hu et al. [43] found that AC can also increase the stiffness, moisture susceptibility, and rutting of the mixture. He recommended that a pavement layer of 6 cm thickness and 18% air void content can provide the best filtration of runoff. AC can be a promising material to enhance the stiffness while absorbing NPS pollution.

## 5. Conclusions

This review paper summarized the literature on porous asphalt mixtures (PAMs) as one of the main components of permeable pavement systems (PPSs), emphasizing the different ways to improve its performance by incorporating different additives. The following points can be drawn out from the study:

- Fillers improve the strength of porous asphalt mixtures; they are adopted in low amounts, which emphasizes their adverse effects on the permeability. The optimum content of fillers should be carefully decided for appropriate permeability and strength.
- The Cantabro test does not consider the stresses due to braking and shear kneading failure that are common in urban areas. Hence, for testing the abrasion resistance of porous asphalt mixtures for urban pavements, the rotating surface abrasion test may provide more realistic results. More testing facilities should be developed that can simulate the actual loading conditions of a porous pavement in cities.
- High-viscosity binder improves the cohesion of the binder, whereas PMB improves the interaction of the binder and aggregates. Both of these binders can improve the resistance of the PAM against abrasion, moisture, and temperature susceptibility. Meanwhile, CRMB can be used if reduction in thermal susceptibility is the main concern.
- Mixes with crumb rubber additive can be used in extreme weather conditions as it reduces the binder’s rigidity at low temperatures, thereby improving crack resistance. At high temperatures, it enhances the stiffness of the binder. However, the addition of crumb rubber reduces the abrasion resistance of mixes.
- Cellulose, glass, and mineral fibers as additions have a high bitumen absorption tendency; they are used to prevent binder draindown, but they do not improve the raveling resistance of PAMs.
- Steel fibers have multiple benefits in enhancing PAM. They not only improve the stability value but also facilitate induction healing in PAM due to their electro-thermal nature. Mixtures with polypropylene and polyester fibers have positive effects on the rutting of the mixture as they act as three-dimensional reinforcement, facilitating more binder stability.
- Combinations of additives and additions were shown to have synergistic benefits, such as nanosilica in combination with fibers; fibers improve the mechanical strength and nanosilica improves the adhesion, stability, and abrasion resistance of the PAM.
- The use of eco-friendly materials in PAM shows promising results. Waste materials like bleaching clay can be utilized in place of limestone filler as it can improve the strength of PAMs. Activated carbon absorbs non-point source gases and, hence, has the potential to mitigate pollution without compromising the abrasion resistance.

For future research, the effect of adding combinations of additives should be studied in detail, as they may have synergistic benefits in the performance of PAMs. Limited literature is available on the incorporation of synthetic fibers in PAMs; therefore, the effects of adding synthetic fibers are still not clear. Nanosilica improves the performance of the PAM, although its effect on the functional characteristics of the PAM is uncertain; thus, it should be analyzed in depth. Additionally, to improve the effectiveness of the implementation of PPSs, the decision-making process should consider that the influence in areas prone to flood can be mapped using a geographic information system (GIS).

## Figures and Tables

**Table 1:** Tests for mechanical resistance and functional performance of porous asphalt mixtures (PAMs).

Test	Standards	Property Tested	Observations
Dry Cantabro test	EN-12697-17, Tex-245-F, T0733-2011	Raveling/abrasion resistance	Developed in the University of Cantabria in 1980s [67] to quantify minimum binder content for PAM design. Also used to measure the raveling resistance. The Cantabro test does not consider the stresses due to braking or shear kneading failure, common in urban areas.
Wet Cantabro test	Spanish NLT 362/92	Moisture sensitivity	Samples are conditioned at 60 °C in water to calculate the abrasion resistance in wet conditions [25].
Rotating surface abrasion test (RSAT)		Stone loss	This test accurately simulates the vehicle tire contact on porous asphalt by application of tangential stresses of a full-sized tire [24,68,69].
Draindown test	AASHTO T305, EN-12697-18, T0732-2011	Binder draindown	To quantify the maximum binder content for PAM design. Virgin binder is unable to prevent draindown. Additives or modifiers are used to improve the stability of the mix [25,26,27,28].
Loaded wheel tracker (LWT) test	AASHTO T324, EN 12697-22	Rutting behavior	Rutting resistance of PAMs reduces considerably due to high moisture, temperature, and loading conditions [70]. Large stone porous mixtures have better rutting resistance [71]
Indirect tensile strength (ITS)	ASTM D 4123, EN 12697-23	Strength	The strength of PAMs varies considerably when additives and modifiers are added.
Tensile strength ratio	AASHTO T283, EN-12697-12	Moisture sensitivity	PAMs have very high moisture susceptibility; nanosilica is added to reduce moisture susceptibility of mixes [72].
Resilient modulus	ASTM D4123	Stiffness	Resilient modulus represents the ability to recover under repeated loads [8]. PAMs are susceptible to temperature; as temperature increases, the stiffness reduces. Additions of diatomite may reduce the thermal susceptibility of PAMs [46]. Increases in the amount and size of crumb rubber usually result in a reduction of resilient modulus [8].
Thermal stress restrained specimen test (TSRST)	AASHTO TP10-93	Low-temperature cracking resistance	In frozen regions, PAMs incorporated with warm-mix additives can be used to reduce cracking resistance [73].
Permeability test	ASTM D2434-68; AASHTO T215-14; ASTM PS 129-01	Permeability coefficient	Permeability depends on the interconnected air voids; constant head tests assume laminar flow which is not the case for PAMs [74]. Fully clogged mixes have negligible permeability.
Air void content	EN 12697-8	Air void content	If the air voids are high, more clogging cycles are required to clog the sample [41,74,75]
Acoustic impedance tube	ASTM 1050-10	Sound absorption	In low-speed vehicles, the noise produced is mainly dependent on the micro texture of the pavement. Macro texture is responsible for noise caused due to high-speed vehicles. The noise reduction depends on the content, size, and distribution of air voids [66,76].
British pendulum tester (BPT)	ASTM E303	Skid resistance	This does not simulate the interaction between the rubber tire and the surface of the pavement as both tests measure the on-spot friction of the pavement [67,77].
Dynamic friction tester (DFT)	ASTM E-1911-98		
Walking friction test (WFT)		Skid resistance	It measures skid between the surface of the pavement and the tire of the vehicle.

**Table 2:** Comparison of performance of additives in PAMs.

Fibers	Type of Additive	Content (%)	AirVoids (%)	Binder	Binder Content (%)	Mesh Size	length (nm)	Cantabro (dry)	Draindown	ITS	TSR	Stiffness	Permeability	Porosity	Rutting	LTCR ^@^
Nanosilica/[81]	Additive	2 (mix)		VB 60/70	5		10–13	O	O	O	O		X	X		
Nanosilica/[81]		4 (mix)		VB 60/70	5.5		20–30		O	O					O	
Nanosilica/[59]	Additive	2 (bit)					10–15			O	O					
Nanosilica/[85]	Additive	4 (bit)							O	O					O	O
Nanosilica/[83]	Additive	2 (bit)	20		5.16										O	
Crumb rubber/[26]	Additive	5 (bit)		PG 64-22	5.50	30		X		O	O		XX	X	X	
Crumb rubber/[26]	Additive	12 (bit)		PG 64-22	7.00	30		XX		O	O		XX	XX	X	
Crumb rubber (additive)/[8]	Additive	10 (bit)	18.6	VB 50/70	6.50	20~200 4~20, 4~200		O		O	XX	O				
Crumb rubber (additive)/[8]	Additive	15 (bit)	18.6	VB 50/70				XX		XX	XX	XX				
Crumb rubber (additive)/[8]	Additive	20 (bit)	18.6	VB 50/70				XX		XX	XX	XX				
Crumb rubber (additive)/[8]	Additive	3, 6, 9 and 12 (bit)	≈22	VB 80/100	5	40		XX				X	X	X		
Crumb rubber (additive)/[89]	Additive	1 (agg)	≈25	SBS	6			O		X	O	O	XX			O
Sasobit^®^/[91]	Warm-mix additive	2.5 (bit)	28.78	PMB 45/80-65	5.20			XX		O	XX		O			
Evotherm/[92]	Warm-mix additive	0.5 (bit)	20.40	PG 76-22	5.70			O	O				O	O	XX	O
Stearic acid amide/[73]	Warm-mix additive	3 (bit)	23.8	SBS				XX				X	O	O	O	O
Diatomite/[16]	Anti-stripping additive	2 (agg)	21	PG76	5.25			XX					O			
Hydrated lime/[16]	Anti-stripping additive	2 (agg)	21	PG76	5			XX					O			
Ordinary Portland cement (OPC) /[16]	Anti-stripping additive	2 (agg)	21	PG76	5			XX					O			

Note: * SBS-modified bitumen; O means improvement; #VB Virgin binder; X refers to no significant differences; @LTCR Low-temperature crack resistance; XX refers to adverse effects by the weight of aggregate.

**Table 3:** Comparison of performance of fibers in PAMs.

Fibers/Reference	Content (%)	Air voids (%)	Binder	Binder Content (%)	Length (mm)	Diameter (μm)	Cantabro (dry)	Cantabro (aged)	Draindown	ITS	TSR	Stiffness	Permeability	Porosity	Rutting	LTCR ^@^
Cellulose/[25]	2.5 (agg)	20.8	HVB	5.10			XX	O	O	-	X	-			-	O
Cellulose/[26]	0.3 (mix)		PG 64-22	5.50	6		XX	-	O	X	O	X	X	XX	O	
Cellulose/[27]	0–0.5	18.6	PMB 45/80	4.70	1.1	45	XX		O	X	X	X	O		O	
Cellulose/[64]	0.1, 0.3, 0.5		SBS *	5	3	200 ± 5			O		O					
Cellulose/[28]	0.25, 0.5, 0.75 (mix)	20%	VB^#^ 50/70	4.50	1.1	45	O		O	XX	XX		XX			
Cellulose/[37]	0.30 (mix)	≈20	AR-80	5.20			X	XX		X	X		XX		O	
Mineral (basalt)/[81]	0.2 (mix)		VB 60/70	5	24	18	XX	XX	O	O	X		XX	X		
Mineral (basalt)/[81]	0.2 (mix)		VB 60/70	6	24	18	XX	XX	O	O	XX		XX	X		
Glass/[81]	0.2 (mix)		VB 60/70	5	12	10	XX	XX	O	O	XX		XX	X		
Glass/[81]	0.2 (mix)		VB 60/70	6	12	10	XX	XX	O	O	X		XX	X		
Polypropylene + glass/[81]	0.3 + 0.1 (mix)		VB 60/70	4.5	12 + 12	2200 + 10			O	O					O	
Polypropylene + glass/[81]	0.3 + 0.1 (mix)		VB 60/70	5.5	12 + 12	2200 + 10			O	O					O	
Polypropylene + glass/[81]	0.3 + 0.1 (mix)		VB 60/70	6	12 + 12	2200 + 10			O	O					O	
Polypropylene/[64]	0.1–0.5		SBS	5	3	25–50			O		O					
Polyester/[25]	2.5 (agg) ^@^	20.1	HVB	5.1			O	O	O	XX	X	XX	XX		O	O
Polyester/[64]	0.1–0.5		SBS	5	3	20 ± 5			O		O					
Polyester/[24]	0.10	≈21	SK70	4.4			O		X							
Mineral/[25]	2.5 (agg)	20.7	HVB	5.1			O	O	O	-	X	-			-	O

Note: * SBS-modified bitumen; O means improvement; #VB Virgin binder; X refers to no significant differences; @LTCR Low-temperature crack resistance; XX refers to adverse effects by the weight of aggregate.

**Table 4:** Comparison of eco-friendly materials with virgin materials. RCA—recycled concrete aggregate.

Material	Type ofAdditive	Content%	Air Voids	Binder	BinderContent (%)	Cantabro (dry)	Draindown	ITS	Moisturesusceptibility	Stiff ness	Permeability	Porosity	Rutting
Waste glass/[35]	Aggregates	94 (agg)	16.5	Pen 60/70	6	X	O	O	O		X		X
RCA/[36]	Aggregates	16 (agg)	19.5	Pen 60/71	6	XX	O	O	O		X		X
Bleaching clay/44[44]	Filler	5 (agg)		PMB 45/80	5.1	XX		O	X	O	XX		
Red mud/[45]	Filler	1.5 (mix)	≈18	SBS	4.8	O			O				
Activated carbon/[103]	Additive	8 (mix)	20		5.95	X	O	X				X	
Activated carbon/[43]	Additive	1–4 (agg)	21	SBS *	6.03	O	O	XX	O				O
RCA/[35]	Aggregates	100 (agg)	19.4	Pen 60/70	5.5	XX	X		O		O		
Reclaimed Tetra pak/[28]	Fiber	0.25–0.75 (mix)	≈20	VB 50/70	4.5	O	O	O			XX		

Note: O means improvement; * SBS-modified bitumen; X refers to no significant differences-; #VB Virgin binder-; XX refers to adverse effects@ by the weight of aggregates.
